# *Bacillus anthracis*-derived edema toxin (ET) counter-regulates movement of neutrophils and macromolecules through the endothelial paracellular pathway

**DOI:** 10.1186/1471-2180-12-2

**Published:** 2012-01-09

**Authors:** Chinh Nguyen, Chiguang Feng, Min Zhan, Alan S Cross, Simeon E Goldblum

**Affiliations:** 1 Southern Arizona Veterans Affairs Health Care Systems, 3601 S 6th Ave, Mail Code 111-1, Building 2, 4th floor, Tucson AZ 85723, USA; 2 Center for Vaccine Development, University of Maryland School of Medicine, 685 West Baltimore St, Health Science Facility 1, Rm 480, Baltimore, Maryland 21201, USA; 3 Department of Epidemiology and Public Health, University of Maryland School of Medicine, 660 West Redwood St, Howard Hall, Rm 114A, Baltimore, Maryland 21201, USA; 4 Mucosal Biology Research Center, University of Maryland School of Medicine, Health Science Facility 2, Room 303D, Baltimore, Maryland 21201, USA

## Abstract

**Background:**

A common finding amongst patients with inhalational anthrax is a paucity of polymorphonuclear leukocytes (PMNs) in infected tissues in the face of abundant circulating PMNs. A major virulence determinant of anthrax is edema toxin (ET), which is formed by the combination of two proteins produced by the organism, edema factor (EF), which is an adenyl cyclase, and protective antigen (PA). Since cAMP, a product of adenyl cyclase, is known to enhance endothelial barrier integrity, we asked whether ET might decrease extravasation of PMNs into tissues through closure of the paracellular pathway through which PMNs traverse.

**Results:**

Pretreatment of human microvascular endothelial cell(EC)s of the lung (HMVEC-L) with ET decreased interleukin (IL)-8-driven transendothelial migration (TEM) of PMNs with a maximal reduction of nearly 60%. This effect required the presence of both EF and PA. Conversely, ET did not diminish PMN chemotaxis in an EC-free system. Pretreatment of subconfluent HMVEC-Ls decreased transendothelial ^14 ^C-albumin flux by ~ 50% compared to medium controls. Coadministration of ET with either tumor necrosis factor-α or bacterial lipopolysaccharide, each at 100 ng/mL, attenuated the increase of transendothelial ^14 ^C-albumin flux caused by either agent alone. The inhibitory effect of ET on TEM paralleled increases in protein kinase A (PKA) activity, but could not be blocked by inhibition of PKA with either H-89 or KT-5720. Finally, we were unable to replicate the ET effect with either forskolin or 3-isobutyl-1-methylxanthine, two agents known to increase cAMP.

**Conclusions:**

We conclude that ET decreases IL-8-driven TEM of PMNs across HMVEC-L monolayers independent of cAMP/PKA activity.

## Background

Anthrax refers to those clinical syndromes caused by the spore-forming, Gram-positive organism, *Bacillus anthracis *[1]. Classically, anthrax presents as one of three syndromes: cutaneous, gastrointestinal, and pulmonary [1]. Pulmonary anthrax is among the most feared of infectious diseases; once clinical symptoms have developed, mortality remains high even with appropriate treatment. Much of the pathogenesis of anthrax is currently attributed to two toxins, each of which is produced from two of three proteins synthesized by the bacillary form of the organism: protective antigen (PA), edema factor (EF), and lethal factor (LF) [1]. PA combines with either LF to form lethal toxin (LT), or with EF to form edema toxin (ET) [1]. LT received its name as it was thought to be the principal virulence determinant responsible for the most deleterious sequelae of anthrax infection [1]. ET was so named as it caused localized edema, *in vivo*, upon subcutaneous injection [1].

The mechanisms through which ET elicits host cell responses are incompletely understood. PA is the receptor binding moiety of the toxin complex. After binding to one of two surface receptors, endothelial marker-8 (TEM-8)/anthrax receptor 1 (ANTXR1) or capillary morphogenesis protein-2 (CMG-2)/anthrax receptor 2 (ANTXR2), PA is cleaved into a 63 kDa fragment by surface proteases, such as furin [2,3]. ANTXR1 is present in the epithelial cells lining the respiratory pathway, skin, and gastrointestinal tract, as well as being selectively upregulated in endothelial cell(EC)s during angiogenesis and tumorigenesis [4]. In contrast, ANTXR2 is ubiquitously expressed in most human tissues [5]. These PA fragments oligomerize into ring-shaped heptamers, to which EF binds [2]. The entire complex then undergoes receptor-mediated endocytosis [2]. This endosome is acidified, resulting in conformational changes, which in turn, permit insertion of the multiprotein complex comprised of EF and the PA cleavage product into the endosomal membrane [2]. EF is then translocated to the cytosol, where it exerts its biological effects [2]. EF is one of four known bacterial products that are intrinsic adenyl cyclases [6]. Its catalytic rate is 100-fold higher than any mammalian equivalent [6]. The current understanding is that most of the effects of EF are due to elevated levels of mislocalized cAMP [1]. ET has been demonstrated to increase cAMP in a variety of cell types, including Jurkat cells, Hela cells, monocytes, and most relevant to the current studies, ECs and polymorphonuclear leukocyte(PMN)s [3,7-9]. cAMP is a ubiquitous secondary messenger with multiple downstream effectors, including protein kinase A (PKA) and protein activated by cAMP (EPAC), a guanine nucleotide exchange factor (GEF) for Ras-related protein 1 (RAP1) [10]. There are two EPAC variants, EPAC1 and EPAC2, each of which has a distinct domain structure and tissue-specific expression [10]. The EPAC1-RAP1 pathway has been implicated in such cellular processes as vascular endothelial (VE)-cadherin-mediated cell-cell adhesion [11-13], integrin mediated adhesion [14], monocyte chemotaxis [15], Ca^2+^-induced exocytosis [16], and Fcγ-receptor mediated phagocytosis [17]. Whether ET might also exert biological effects independent of cAMP is unknown.

Highly purified, recombinant ET is lethal to mice [18] at lower doses than is LT [19]. Curiously, edema was absent in these mice at the microscopic level [18]. ET suppresses the T-lymphocyte secretion of the PMN chemoattractant, interleukin (IL)-8 [20]. ET also impairs PMN phagocytosis and superoxide production [21]. In EC-free systems, investigators have demonstrated that ET increases PMN chemotaxis [22], whereas others have shown an inhibitory effect [9]. Of relevance to the current report, ET also decreases EC chemotaxis [7].

In 2001, renewed interest in pulmonary anthrax was generated when 11 bioterrorism-related cases were described [23,24]. A unifying feature of these cases was a normal to slightly elevated circulating leukocyte count in the face of relatively high levels of bacteremia [24]. Although circulating PMNs were abundant, lung tissues from these patients were notable for a lack of intra-alveolar inflammatory infiltrates [25]. The pleural fluid of several patients contained scant PMNs. Similarly, in African Green Monkeys exposed to anthrax spores, the pulmonary interstitium was expanded by fibrin and edema, but contained few PMNs [26]. These combined data suggest an impaired delivery of circulating PMNs to extravascular sites of infection. Since PMNs are an essential host defense against bacterial infection, a survival advantage would be conferred to any infecting organism that could disable these phagocytic cells.

From its name, most observers would intuit that ET increases edema formation, i.e., the paracellular passage of fluid and macromolecules. However, agents that increase intracellular cAMP are known to enhance EC-EC adhesion, tighten the paracellular pathway, and promote barrier integrity [11,27-32]. He et al found that basal levels of cAMP are necessary to maintain barrier function under resting conditions [30]. Multiple investigators have demonstrated that pharmacologic agents which increase cAMP or behave as cAMP analogues in ECs enhance barrier function [11,27,28,31-33]. Prior studies which have looked at ET effects on PMN chemotaxis have done so in EC-free systems, and therefore did not examine whether ET might impair PMN migration to target tissues through an effect on the endothelium. In the current study, we have defined a novel mechanism through which a bacteria-derived toxin, ET, may indirectly, through the counter-regulation of the endothelial paracellular pathway, impair extravasation of PMNs into tissues.

## Results

### ET protects against IL-8-stimulated transendothelial migration (TEM) of PMNs

**Figure 1:**
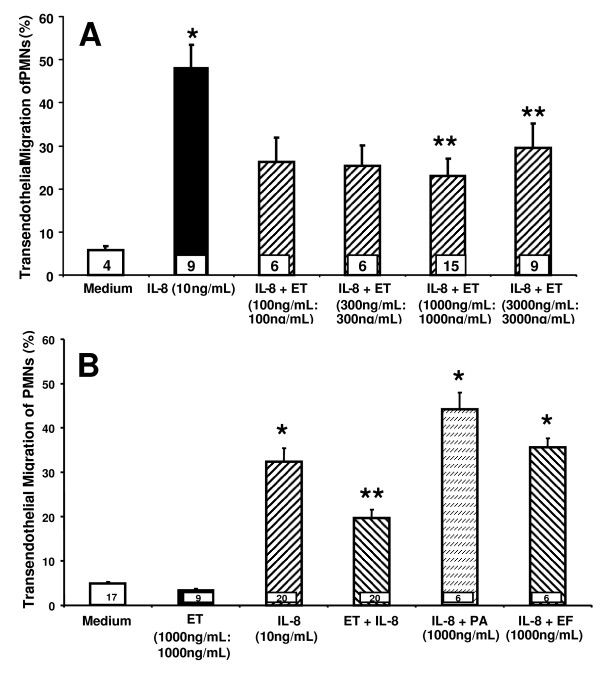
**Effect of ET on the TEM of PMNs**. (**A**) Human microvascular endothelial cells from the lung (HMVEC-Ls) cultured to confluence in assay chambers were exposed for 4 h to either increasing concentrations of ET at the indicated doses each of EF and PA (EF:PA) or medium alone. (**B**) HMVEC-L monolayers cultured to confluence in assay chambers were exposed for 4 h to medium, ET (1000 ng/mL:1000 ng/mL), EF (1000 ng/mL), or PA (1000 ng/mL). These same HMVEC-L monolayers were then inserted into the wells of 24-well plates containing either IL-8 (10 ng/mL) or medium alone, after which calcein-AM-labeled PMNs were added to the upper compartment of each chamber. After 2 h, each lower compartment was fluorometrically assayed. Each vertical bar represents mean (+/- SEM) TEM of PMNs (%). The n for each group is indicated in each bar. * indicates significantly increased compared to the simultaneous medium controls at *p *< 0.05. ** indicates significantly decreased compared to the IL-8 stimulus alone at *p *< 0.05.

Since ET directly stimulates ECs to increase cAMP [7], which in turn, enhances endothelial barrier integrity [11,27-32], we asked whether ET might decrease TEM of PMNs. Pretreatment of monolayers of human microvascular endothelial cells of the lung (HMVEC-Ls) with ET decreased IL-8-stimulated TEM by ~ 60% (Figure 1A). Neither EF nor PA alone were able to reproduce the ET effect (Figure 1B). For these calculations, total fluorescence associated with PMNs placed in each upper compartment represented 100% migration while % migration was calculated as fluorescence in the lower compartment/fluorescence in the upper compartment × 100%.

### ET acts at the level of the EC to decrease IL-8-driven TEM of PMNs

**Figure 2:**
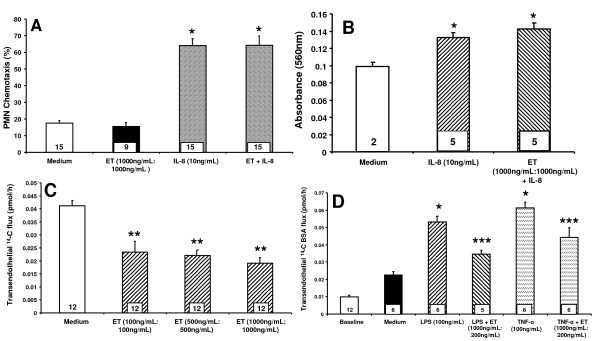
**ET effect on IL-8-driven TEM of PMNs is due to a direct effect on ECs**. (**A**) Naked filters mounted on chemotaxis chambers were placed into wells containing either medium or IL-8 (10 ng/mL), after which calcein-AM-labled PMNs, suspended in medium containing ET (1000 ng/mL:1000 ng/mL) or medium alone, were added to each upper compartment. After 2 h, the contents of each lower compartment were fluorometrically assayed. Each vertical bar represents mean (+/- SEM) chemotaxis of PMNs (%). (**B**) Naked filters were mounted in modified Boyden chemotaxis chambers in which the lower compartment contained either medium or IL-8 (10 ng/mL). PMNs, suspended in medium containing ET or medium alone, were added to each upper compartment. After 0.5 h, the filter was removed, fixed, washed, stained with crystal violet, washed, and the top surface of each filter scraped free of cells. The crystal violet was then extracted and absorbance measured at 560 nm. Each vertical bar represents mean (+/- SEM) absorbance at 560 nm. (**C**) HMVEC-Ls were seeded at a density of 1.0 × 10^5 ^cells/assay chamber and cultured overnight prior to treatment for 6 h with either medium or increasing concentrations of ET. Each vertical bar represents mean (+/- SE) transendothelial ^14 ^C-BSA flux. (**D**) HMVEC-Ls cultured to confluence in assay chambers were treated for 6 h with medium, TNF-α (100 ng/mL), TNF-α in the presence of ET (1000 ng/mL:200 ng/mL), LPS (100 ng/mL), or LPS + ET (1000 ng/mL:200 ng/mL). Each vertical bar represents mean (+/- SEM) transendothelial flux of ^14 ^C-BSA. The n for each group is indicated in each bar. * indicates significantly increased compared to the simultaneous medium controls at *p *< 0.05. ** indicates significantly decreased compared to the simultaneous medium control at *p *< 0.05. *** indicates significantly decreased compared to either TNF-α or LPS alone at *p *< 0.05.

Since ET decreased the TEM of PMNs (Figure 1A), we asked whether it acted directly on PMNs or indirectly via the EC response. When PMNs were co-incubated with ET in the absence of ECs, ET at the same concentration that impaired TEM (1000 ng/mL:1000 ng/L) did not decrease IL-8-driven PMN chemotaxis compared to medium controls (Figure 2A). These data indicate that the ability of ET to diminish TEM of PMNs cannot be explained through a direct effect on PMNs. Since these PMNs were preloaded with the fluoroprobe, calcein-AM, a known intracellular Ca^2+^-binder [34], and the host response to ET is calmodulin- and Ca2 + -dependent [1,2,8,22], we asked whether calcein-AM might diminish PMN responsiveness to ET. The impact of ET on IL-8 driven chemotaxis of unlabeled PMNs was assessed. In these studies, IL-8 increased PMN chemotaxis ~ 1.4-fold compared to the simultaneous medium controls (Figure 2B). The addition of ET did not alter PMN chemotaxis compared to PMNs incubated with IL-8 alone. These data confirm those generated in our studies with calcein-AM-labeled PMNs (Figure 2A) and further support exclusion of a direct ET effect on PMNs.

To establish whether the ability of ET to decrease IL-8-driven TEM of PMNs was mediated indirectly through the EC response, we measured the effect of ET on movement of a permeability tracer across the endothelia. In a subconfluent HMVEC-L monolayers, where the average baseline transendothelial ^14 ^C-albumin flux was 0.0256 (+/- 0.0147) pmol/h, ET, at increasing concentrations, dose-dependently decreased mean (+/- SEM) transendothelial ^14 ^C-albumin flux compared to the simultaneous medium controls (Figure 2C). ET concentrations as low as 100 ng/mL:100 ng/mL diminished transendothelial ^14 ^C-albumin flux. These data indicate that ET restricts passage of macromolecules through the same endothelial paracellular pathway through which PMNs migrate. To provide additional evidence that ET decreases IL-8-driven TEM of PMNs through the EC response, we tested whether ET might protect against agonist-induced barrier disruption. In confluent HMVEC-Ls where the mean (+/- SEM) baseline transendothelial ^14 ^C-albumin flux was 0.01 (+/- 0.006) pmol/h, both human recombinant tumor necrosis factor (TNF)-α and bacterial lipopolysaccharide (LPS), each at 100 ng/mL, increased ^14 ^C-albumin flux > 2-fold compared to the simultaneous medium controls (Figure 2D). When LPS and TNF-α were coadministered with ET at 1000 ng/mL:200 ng/mL, the increase in transendothelial ^14 ^C-albumin flux in response to either LPS or TNF-α was decreased by ≥ 60% and ~ 45%, respectively, compared to albumin flux in response to each respective agonist alone (Figure 2D). These data indicate that ET provides partial protection against both endogenous host and exogenous bacteria-derived mediators of endothelial barrier disruption through its action on ECs.

### The effect of ET on IL-8 driven TEM of PMNs is PKA-independent

**Figure 3:**
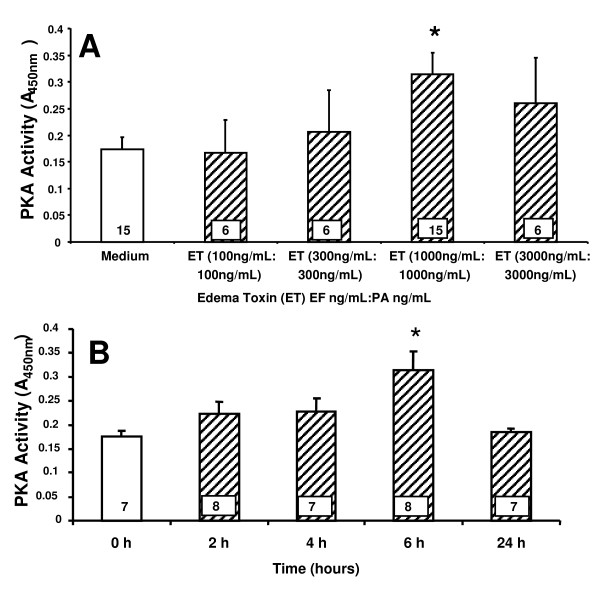
**ET activates PKA in HMVEC-Ls**. HMVEC-Ls were seeded onto 10 cm plates and allowed to reach 80-90% confluence prior to (**A**) 6 h exposure to increasing doses of ET, or (**B**) increasing exposure times with ET (1000 ng/mL:1000 ng/mL). Lysates were collected and PKA activity assayed by ELISA. Each vertical bar represents mean (+/- SEM) absorbance at 450 nm. The n for each group is indicated in each bar. * indicates significantly increased compared to the simultaneous medium controls at *p *< 0.05.

**Figure 4:**
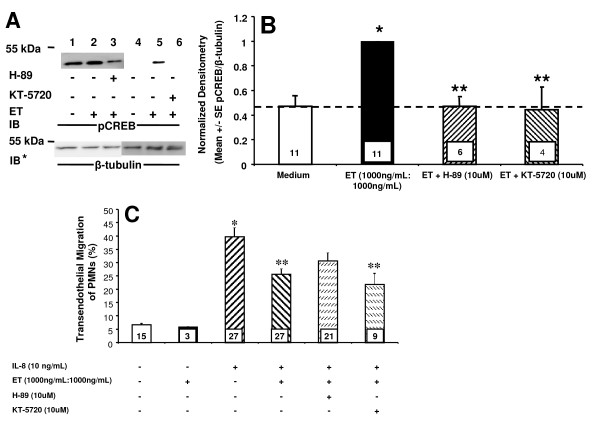
**ET Inhibition of TEM in the Presence of PKA Inhibitors**. (**A**) HMVEC-Ls were preincubated in the presence (+) or absence (-) of H-89 (10 μM) or KT-5720 (10 μM), respectively, before being treated with ET (1000 ng/mL:1000 ng/mL) for 6 h and lysed. The lysates were processed for pCREB immunoblotting. To control for protein loading and transfer, blots were stripped and reprobed for β-tubulin. IB, immunoblot, IB*, immunoblot after stripping. (**B**) The pCREB signals in each blot described in (**A**) were quantified by densitometry of pCREB and normalized to β-tubulin signal in the same lane in the same blot. (**C**) HMVEC-Ls cultured to confluence in assay chambers were pretreated with medium, H-89 (10 μM) or KT-5720 (10 μM), after which they were treated for 4 h with medium, ET, ET with H-89, or ET with KT-5720. The HMVEC-L monolayers were then inserted into wells containing either medium or IL-8 (10 ng/mL), after which calcein-AM-labeled PMNs were added to the upper compartment of each chamber. After 2 h, the contents of each lower compartment were fluorometrically assayed. Each vertical bar represents mean (+/- SEM) TEM of PMNs (%). The n for each group is indicated in each bar. * indicates significantly increased compared to the simultaneous medium only controls at *p *< 0.05. ** indicates significantly decreased compared to IL-8 alone at *p *< 0.05.

Since ET is an adenyl cyclase that increases cAMP, we asked whether the ability of ET to diminish TEM of PMNs might be mediated through EC-generated PKA. First, ET was tested for its ability to increase PKA activity in HMVEC-Ls. ET at 1000 ng/mL:1000 ng/mL, increased PKA activity (Figure 3A). When ECs were exposed for increasing times (0-24 h) to a fixed concentration of ET (1000 ng/mL:1000 ng/mL), PKA activity was increased at 6 h, returning to basal levels at ≤ 24 h (Figure 3B). Two structurally dissimilar PKA inhibitors, H-89 and KT-5720, were then tested for their ability to counteract the ET effect on TEM. To confirm that H-89 and KT-5720 impaired PKA activity in HMVEC-Ls, we examined ET-induced phosphorylation of cAMP response element-binding protein (CREB), a direct PKA substrate [35]. Initially, phospho-CREB (pCREB) signal was normalized to total CREB. However, stripping of the anti-pCREB antibody was incomplete and inconsistent. Consequently, pCREB was normalized to β-tubulin. H-89 and KT-5720 each diminished ET-induced CREB phosphorylation (Figure 4A, lanes 3 vs 2, 6 vs 5). Quantitative densitometry was performed on each of these same blots. H-89 and KT-5720 both completely blocked phosphorylation of CREB normalized to β-tubulin compared to the simultaneous medium controls (Figure 4B), indicating their effectiveness as inhibitors of PKA in HMVEC-Ls. In these experiments, IL-8 (10 ng/mL) increased TEM of PMNs ~ 4-fold when compared to simultaneous medium controls (Figure 4C). Pretreatment of ECs with either H-89 (10 μM) or KT-5720 (10 μM) alone had no effect on TEM in the presence or absence of IL-8 (data not shown). Pretreatment of ECs with ET (1000 ng/mL:1000 ng/mL) decreased IL-8-driven TEM of PMNs by ~ 45%. H-89 and KT-5720 each failed to reverse the ET effect; i.e., the effect of either agent co-administered with ET was not significantly different than ET alone (Figure 4C). Although H-89 and KT-5720 each completely blocked ET-induced increments in PKA activity as measured by pCREB/β-tubulin ratios (Figure 4B), these same inhibitors had no impact on the ET-induced reduction of TEM (Figure 4C). Taken together, these data do not support a PKA-mediated ET effect on TEM.

### Forskolin (FSK) and 3-isobutyl-1-methylxanthine (IBMX) fail to reproduce the ET effect on IL-8-driven TEM of PMNs

**Figure 5:**
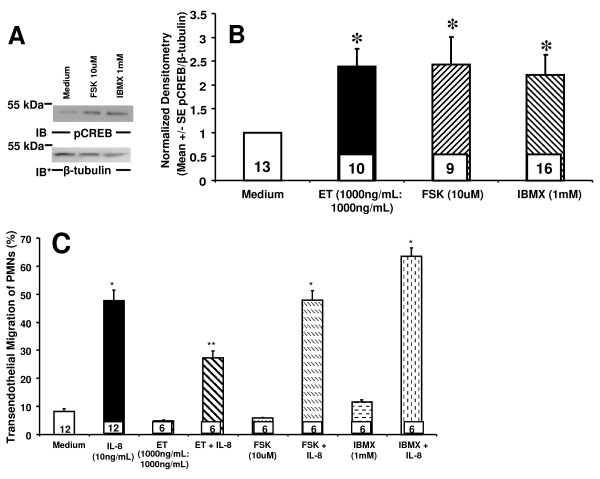
**Agents that increase intracellular cAMP do not reproduce the ET effect on IL-8-driven TEM of PMNs**. (**A**) HMVEC-Ls were treated for 6 h with ET (1000 ng/mL:1000 ng/mL), FSK (10 μM), IBMX (1 mM), or medium alone, and lysed. The lysates were processed for pCREB immunoblotting. To control for protein loading and transfer, blots were stripped and reprobed for β-tubulin. IB, immunoblot, IB*, immunoblot after stripping. (**B**) The pCREB signals in each blot described in (**A**) were quantified by densitometry and normalized to β- tubulin signal in the same lane in the same blot. (**C**) HMVEC-Ls cultured to confluence in assay chambers were treated for 4 h with medium, ET, FSK, or IBMX. These same chambers were then inserted into wells of 24-well plates containing either medium or IL-8 (10 ng/mL), after which calcein-AM-labeled PMNs were added to the upper compartment of each chamber. After 2 h, the contents of each lower compartment were fluorometrically assayed. Each vertical bar represents mean (+/- SEM) TEM of PMNs (%). The n for each group is indicated in each bar. * indicates significantly increased compared to the simultaneous medium controls at *p *< 0.05. ** indicates significantly decreased compared to IL-8 alone at *p *< 0.05.

To provide further evidence that ET does not decrease TEM of PMNs through cAMP or PKA activity, two distinct interventions known to increase cAMP, FSK and IBMX, each were introduced. To confirm that FSK and IBMX increased PKA activity in HMVEC-Ls, we first examined FSK- and IBMX- stimulated phosphorylation of CREB at 6 h (Figure 5A). FSK (10 μM) and IBMX (1 mM) each increased phosphorylation of CREB normalized to β-tubulin when compared to the simultaneous medium control (Figures 5B). Previous investigators have demonstrated that FSK and IBMX cause maximal increases of cAMP at 0.5 h with a decrease by 4 h [36]; in our studies, phosphorylation of CREB normalized to β-tubulin was elevated but not significantly different from the effect at the later time point (Additional File 1: Figure S1A, B). Next, we investigated the effects of FSK and IBMX on IL-8-driven TEM. In these experiments, IL-8 (10 ng/mL) increased TEM of PMNs ~ 6-fold compared to simultaneous medium control (Figure 5C). Pretreatment of ECs with ET decreased TEM of PMNs by ~ 50%. Neither FSK nor IBMX could reconstitute the ET effect on IL-8 driven TEM of PMNs, either at 0.5 h (Additional File 1: Figure S1C) or at 4 h (Figure 5C). Although FSK and IBMX each upregulated PKA activity comparable to that seen after ET treatment (Figure 5B), none could decrease TEM (Figure 5C). Again, these combined data do not support a cAMP/PKA-dependent mechanism through which ET inhibits TEM of PMNs.

## Discussion

In our studies, we have found that ET decreases IL-8-driven TEM of PMNs across human lung microvascular endothelia. We asked whether the observed ET effect could be attributed to action on either the PMN and/or endothelium. We found that ET blocked TEM even when PMNs were not directly exposed to ET (Figure 1A) and required the presence of both EF and PA (Figure 1B). At the same concentrations, ET did not inhibit PMN chemotaxis in an EC-free system (Figure 2A, B). In contrast, we found that ET decreased ^14 ^C-albumin flux across preconfluent endothelia (Figure 2C). Further, ET attenuated the increase in ^14 ^C-albumin flux provoked by both endogenous (TNF-α) and exogenous (LPS) mediators of barrier disruption (Figure 2D). Prior inhibition of PKA with H-89 or KT-5720 did not reverse the ET effect on TEM (Figure 4C), and agents demonstrated to elevate intracellular levels of cAMP in HMVEC-Ls (Figure 5A, B, Additional File 1: Figure S1A, B) could not reconstitute the ET effect (Figure 5C, and Additional File 1: Figure S1C). These combined data indicate that ET diminishes TEM of PMNs at the level of the endothelial paracellular pathway and does so independent of via cAMP/PKA activity.

Several studies have examined the direct effect of ET on *in vitro *PMN function. O'Brien et al found that ET inhibited PMN phagocytosis of opsonized *B. anthracis *[21]. Pretreatment of PMNs with ET profoundly reduced superoxide production in response to either LPS or muramyl dipeptide. Crawford et al demonstrated that ET impaired PMN NADPH oxidase activation and downstream N-formyl-methionine-leucine-phenylalanine (fMLP)-induced superoxide production [37]. Taken together, these studies indicate that ET down-regulates PMN phagocytic and oxidative functions. Other studies have focused on the impact of ET on PMN chemotaxis and migration [9,22]. In the current studies, ET did not alter the PMN chemotactic response to IL-8 in an EC-free system (Figure 2A). To address concerns that calcein is a Ca^2+^-binder and would interfere with any Ca^2+^-mediated ET effect, these experiments were performed in the absence of the fluoroprobe. Even in the absence of calcein, ET had no effect on IL-8 chemotaxis of PMNs (Figure 2B). Chemotaxis was not as vigorous in the latter experiment, and this may be secondary to differences in methodology; mainly the use of a modified Boyden chambers, a shorter incubation time, as well as a different means of measuring PMN migration.

Wade et al found that ET stimulated directed neutrophil migration without having any effect on unstimulated random migration [22]. They also found that although ET increased cAMP in PMNs, the absolute level of that increase was < 1% of that caused by the *Bordetella pertussis *toxin. In contrast, Szarowicz et al found that ET reduces chemoattractant-stimulated PMN actin assembly, chemokinesis, chemotaxis and polarization [9]. In PMNs, ET provoked a > 50-fold increase in cAMP and a 4-fold increase in PKA phosphorylation. The differences between our findings and these other reports may be attributed to dissimilar techniques. For instance, Wade et al measured chemotaxis of PMNs preincubated for 1 h with ET in an agarose-gel based system, both of which were EC-free [22], whereas Szarowicz's group utilized video microscopy to study adherence of PMNs preincubated for 2 h with ET to a fibronectin-coated surface [9]. To our knowledge, none of these previous reports studied PMN migration in the context of the endothelial paracellular pathway. Another potential explanation for these disparities may be due to differences in potency of various EF preparations and their abilities to generate cAMP. Of note, the EF preparation offered by List Biologics is the least potent (personal communication, Dr. Erik Hewlett, University of Virginia, Charlottesville).

Far less is known about the direct effect of ET on ECs. Hong et al demonstrated that ET reorganizes the cytoskeleton and inhibits chemotaxis of human microvascular ECs [7]. Tessier's group found that ET induces a gradual increase in transendothelial electrical resistance (TEER) across human umbilical vein EC monolayers cultured on collagen-coated inserts. They concluded that ET-induced edema could not be accounted for by the direct effect of ET on the endothelium [38]. Of interest, in our experimental systems for both TEM of PMNs and transendothelial ^14 ^C-albumin flux, the ECs were similarly cultured on collagen-impregnated filters. Although Tessier et al studied TEER, their experiments did not include transendothelial flux of a permeability tracer or TEM of PMNs.

ET is an intrinsic adenyl cyclase that increases cAMP [1]. Data exists to support a cAMP-mediated mechanism underlying the ET effect on TEM of PMNs. Moy et al found that cAMP agonists attenuated the ability of thrombin to increase permeability [27]. Similarly, Fukuhara et al found that cAMP agonists decreased cell permeability and enhanced vascular EC-EC adhesion [11]. In ECs, cAMP targets multiple downstream signaling molecules that might promote endothelial barrier integrity, including PKA [39] and EPAC1 [40,41].

One key effector of cAMP is PKA [10]. PKA has been shown to inhibit myosin-based contractility through phosphorylation of myosin-light-chain-kinase, thereby decreasing its activity [10]. PKA also inhibits RhoA activity, stabilizes microtubules, reorganizes cortical actin and strengthens tight junctions through phosphorylation of vasodilator stimulated protein (VASP) [10]. In our studies, we found that ET activates PKA in HMVEC-Ls in a dose- and time- dependent manner (Figure 3A, B). Although ET increases EC PKA activity, its inhibitory effect on TEM could not be ascribed to PKA activity. Two structurally dissimilar pharmacologic inhibitors of PKA, H-89 and KT-5720, each failed to attenuate the ET-induced decrease in IL-8-driven TEM of PMNs (Figure 4C). Further, we were unable to reproduce the ET effect on TEM with either of two structurally and functionally distinct pharmacologic agents each known to increase cAMP, FSK or IBMX (Figure 5C). Taken together, these data indicate that the mechanism through which ET counter-regulates IL-8-driven TEM of PMNs cannot be explained solely through cAMP/PKA activation.

Another downstream target for cAMP is EPAC1, which is a GEF for the ras GTPase, RAP1 [10]. Like PKA activity, the EPAC1-RAP1 pathway also enhances endothelial barrier function [11,12,42-44]. The EPAC1-specific analog 8CPT-2'O-Me-cAMP, which directly activates EPAC1 while bypassing PKA, has been shown to decrease permeability of endothelial cell monolayers, an effect which is ablated by prior siRNA-induced EPAC1 knockdown [12]. Birukova et al [44] and Fukuhara et al [11] both demonstrated that activation of EPAC1 attenuated thrombin-induced increases in permeability. As in the case of PKA, the mechanism(s) by which EPAC1 improves barrier function is still being elucidated. Potential EPAC1 targets include activation of VASP, as well as activation of ARAP3, which in turn is a GEF for RhoA, and vinculin, which supports EC-EC adherens junctions through association with α-catenin [10]. As PKA inhibition did not impair the ET effect on TEM (Figure 4C), one potential pathway is through EPAC1-RAP1 and its effectors.

Since ET evokes biological responses in both PMNs and ECs, it was unclear as to whether the ability of ET to regulate TEM of PMNs could be ascribed to its impact on PMNs, ECs, or both. Although prior studies had demonstrated that ET directly influenced PMN chemotaxis, in our experiments, it did not (Figure 2A). Further, ET diminished TEM of PMNs never exposed to ET (Figure 1A). Finally, not only did ET decrease the paracellular movement of PMNs (Figure 1A), but of a permeability tracer as well (Figure 2B, C). These combined data indicate that ET counter-regulates PMN diapedesis exclusively through its effects on the endothelium. Further support of this concept is offered by Wittchen et al, who reported direct activation of RAP1 in EC monolayers decreased both their permeability as well as TEM of leukocytes [43].

## Conclusions

In conclusion, we have found that anthrax-derived ET impedes IL-8 driven movement of PMNs across an EC monolayer, as well as attenuates the increase of transendothelial ^14 ^C albumin flux induced by TNF-α and LPS, likely as a direct effect of ET on EC-EC adhesion. This ability to counter-regulate paracellular pathway function could not be ascribed to cAMP/PKA activity. Whether this novel pathophysiology for anthrax can be extended to other pathogenic bacteria and their toxins requires further study.

## Methods

### Reagents

H-89 and KT-5720 in-solution were purchased from Calbiochem (Gibbstown, NJ). LPS derived from *E. coli *0111:B4, FSK, and IBMX were purchased from Sigma (St. Louis, MO). EF and PA were purchased from List Biologics (Campbell, CA). Human TNF-α was purchased from R&D Systems, Inc. (Minneapolis, MN). Biotinylated rabbit monoclonal anti-pCREB, murine monoclonal anti-CREB antibodies, horseradish peroxidase (HRP)-conjugated streptavidin, HRP-conjugated goat anti-rabbit IgG, and HRP-conjugated horse anti-murine IgG antibodies were purchased from Cell Signaling Technology (Danvers, MA). Unconjugated murine monoclonal anti-β-tubulin was purchased from Invitrogen (Carlsbad, CA).

### EC culture

Human microvascular endothelial cells from the lung (HMVEC-Ls), purchased from Promocell (Heidelberg, Germany) were cultured in EC growth medium MV-2 (Promocell) containing 5% fetal bovine serum, human recombinant epidermal growth factor (5 ng/mL), human recombinant insulin-like growth-factor-1 (20 ng/mL), human basic fibroblast growth factor (10 ng/mL), vascular endothelial growth factor (0.5 ng/mL), hydrocortisone (0.2 μg/mL), ascorbic acid (1 μg/mL), gentamicin (30 μg/mL), and amphotericin B (15 ng/mL) [45]. Only ECs in passages 6-8 were studied.

### Preparation and fluorescent labeling of PMNs

Whole peripheral blood from healthy human volunteers was collected under a protocol approved by the University of Maryland, Baltimore, Institutional Review Board, into acid citrate dextran (Sigma) solution, and PMNs were isolated by dextran erythrocyte sedimentation and density gradient centrifugation through Ficoll-Hypaque (Sigma) as previously described [46]. PMNs were resuspended in Hank's balanced salt solution (HBSS) without divalent cations (HBSS^-^) at 5 × 10^5 ^PMNs/ml and were incubated with 5 μM calcein-AM (Invitrogen) for 30 min at 37°C [46]. PMNs were washed three times with HBSS^- ^after which their purity was > 95% and viability 98% by trypan blue dye exclusion. PMNs were resuspended in HBSS with divalent cations (HBSS^+^) immediately prior to use.

### Assay for TEM of PMNs

TEM of PMNs was assayed as previously described [46]. Briefly, gelatin-impregnated polycarbonate filters (13 mm diameter, 3 μm pore size; Nucleopore, Pleasanton, CA) were mounted in polysterene chemotactic chambers (ADAPS, Dedham, MA), and sterilized overnight with UV irradiation. These chambers, which serve as the upper compartment for each assay chamber, were inserted into the wells of 24-well plates, each well serving as the lower compartment of the assay chamber and containing 1.5 mL of medium. Each upper compartment was seeded with 2.0 × 10^5 ^HMVEC-Ls/chamber in 0.5 mL and cultured to confluence (48 h, 37°C, 5% CO_2_). The EC monolayers cultured on filter supports were treated for 4 h with either ET at increasing concentrations or medium alone. In other experiments, the EC monolayers were treated for either 0.5 h or 4 h with either FSK (10 μM), IBMX (1 mM), or medium alone. These same chambers were then inserted into wells containing IL-8 (10 ng/mL) or medium alone. Calcein-AM-labeled PMNs (5 × 10^5 ^cells/well) were introduced into the upper compartments of assay chambers, incubated for 2 h at 37°C, after which time the contents of each lower compartment were fluorometrically assayed in a Thermo Scientific Fluoroskan Ascent fluorometer (excitation 485 nm, emission 530 nm). The fluorescence of 5 × 10^5 ^calcein-AM labeled PMNs was used to generate total fluorescence. % TEM was expressed as fluorescence signal in the lower chamber/total fluorescence signal in the upper compartment × 100%.

### Chemotaxis of PMNs

Chemotaxis of PMNs was assayed as described [47]. Briefly, gelatin-impregnated polycarbonate filters were mounted in chemotactic chambers, and the chambers inserted into the wells of 24-well plates containing IL-8 (10 ng/mL) or medium alone, as described above. Calcein-AM-labeled PMNs (5 × 10^5 ^cells/well) were suspended in either medium alone versus medium containing increasing concentrations of ET before being placed into the upper compartment of assay chambers and incubated for 2 h at 37°C. The lower compartment was then sampled and fluorometrically assayed. The fluorescence of 5 × 10^5 ^calcein-AM-labeled PMNs was used to generate total fluorescence. % chemotaxis was then expressed as fluorescence signal in the lower chamber/total fluorescence signal in the upper compartment × 100%. In other experiments, unlabeled PMNs were introduced into the upper compartment of a modified Boyden chemotaxis chamber (Neuroprobe Inc., Gaithersburg, MD) while each lower compartment contained IL-8 (10 ng/mL). After 0.5 h, filters were removed, fixed, and washed. PMNs adherent to filters were stained with crystal violet, washed again, and the top surface of each filter scraped free of stained PMNs. The crystal violet was then extracted from each filter with 0.1 M citric acid in 50% ethanol for 5 min and the A_560 nm _of extracts measured, as described [48].

### Assay of transendothelial albumin flux

Transendothelial ^14 ^C-bovine serum albumin (BSA) flux was assayed as described [45], with minor modifications. Briefly, gelatin-impregnated polycarbonate filters (13 mm diameter, 0.4 μm pore size) were mounted on chemotactic chambers, sterilized, and inserted into the wells of 24-well plates. HMVEC-Ls were cultured in the upper compartment of each assay chamber. The baseline barrier function of each monolayer was established by introducing an equivalent concentration of the permeability tracer, ^14 ^C-BSA (1.1 pmol, i.e., 4800-6200 dpm/0.5 ml) (Sigma; St. Louis, MO), to each upper compartment for 1 h, after which 0.5 ml from the lower compartment was mixed with 4.5 ml of Optifluor Scintillation fluid (Packard Instruments, Downers Grove, IL) and counted in a liquid scintillation counter (Beckman, Fullerton, CA). In selected experiments, ECs were seeded at 1 × 10^5 ^cells/chamber and cultured overnight to 80-90% confluence. Here, monolayers were cultured to subconfluence because baseline permeability in postconfluent monolayers was so low as to make detection of any further decreases difficult to measure in our assay system. The monolayers were then exposed for 6 h to increasing concentrations of ET, each with a fixed ratio of EF to PA of 1 ng/mL:1 ng/mL, or medium alone, after which transendothelial ^14 ^C-BSA flux was assayed. In other experiments, ECs were seeded at 2 × 10^5 ^cells/chamber and cultured to confluence over 48 h. The baseline barrier function of each monolayer was established and only those chambers which retained ≥ 97% of the permeability tracer were studied. The monolayers were then exposed for 6 h to LPS (100 ng/mL), TNF-α (100 ng/mL), either LPS or TNF-α in the presence of increasing concentrations of ET, with a fixed ratio of EF to PA of 5 ng/mL:1 ng/mL, or medium alone. Transendothelial ^14 ^C-BSA flux was again assayed and was expressed in pmol/h.

### ELISA for PKA activity

PKA activity was measured in HMVEC-Ls using an ELISA (Stressgen, Plymouth Meeting, PA) for the screening of activators and inhibitors of PKA, according to the manufacturer's instructions [49]. Briefly, HMVEC-Ls were seeded into 10 cm dishes and cultured to 80-90% confluence. The pharmacological agent of interest was added for the indicated time, after which cells were lysed. The lysates were then added to the microtiter plate, whose wells were pre-coated with a substrate that can be phosphorylated by PKA. ATP was added and the reaction was allowed to proceed for 90 min at 30°C. The contents of each well were decanted before rabbit polyclonal antibody specific for the phosphorylated PKA substrate was added. The plate was incubated for 60 min at room temperature, washed four times, incubated for 30 min with HRP-conjugated anti-rabbit IgG, again washed, and incubated with tetramethylbenzidine (TMB) substrate. After 1 h, the stop solution was added and A_450 nm _measured. A standard curve was generated using purified PKA provided by the manufacturer.

### pCREB, CREB, and β-tubulin immunoblotting for PKA activity

Postconfluent HMVEC-Ls were exposed to ET (1000 ng/mL:1000 ng/mL), ET + H-89 (10 μM), ET + KT-5720 (10 μM), FSK (10 μM), IBMX (1 mM), or medium alone, after which they were lysed with ice-cold modified radioimmunoprecipitation assay buffer, containing 50 mM Tris-HCl, pH 7.4, 1% Nonidet P-40, 0.25% sodium deoxycholate, 150 mM NaCl, 1 mM EGTA, 100 mg/ml type-1 DNase, 1 mM sodium orthovanadate, 1 mM NaF, 1 mg/ml pepstatin A, 10 mM pyrophosphate, and 1 mM phenylarsine oxide (all purchased from Sigma), and 1 tablet of complete protease inhibitor mixture (Roche Applied Science) per 20 ml of lysate as described [50]. The lysates were centrifuged, and the supernatants were assayed for protein concentration with a Bradford protein assay kit (Bio-Rad). The samples were resolved by 8-16% gradient SDS-PAGE and transferred onto PVDF membranes. The blots were blocked with membrane blocking solution (Zymed Laboratories Inc., San Francisco, CA) and were incubated with biotinylated rabbit anti-pCREB antibodies (Cell Signaling), followed by streptavidin HRP (Cell Signaling), after which they were developed with enhanced chemoluminescence (ECL). To control for protein loading and transfer, the blots were stripped and reprobed with either murine anti-CREB and/or murine anti-β-tubulin (Invitrogen), and each pCREB band was normalized to total CREB and/or β-tubulin signal in the same lane on the same blot.

### Statistics

One-way analysis of variance, followed by *post hoc *comparisons using Tukey-Kramer's multiple pairwise comparison test, was used to compare the mean responses among experimental and control groups for all experiments. SAS 9.2 was used for the analyses (SAS Institute Inc., Cary, NC, USA). A *p *value of < 0.05 was considered significant.

## Authors' contributions

CN was responsible for acquisition of data and writing the manuscript. CF assisted in the isolation of neutrophils, participated in the design of the study and assisted in drafting the manuscript. MZ performed the statistical analysis. AC participated in study design, drafting the manuscript, and revising it critically. SG participated in study design, drafting the manuscript, and revising it critically. All authors read and approved the final manuscript.

## Supplementary Material

Additional file 1 **Figure S1**. FSK and IBMX do not reproduce the ET effect on IL-8-driven TEM of PMNs at 0.5 h. (**A**) HMVEC-Ls were treated for 0.5 h with FSK (10 μM), IBMX (1 mM), or medium alone, and lysed. The lysates were processed for pCREB immunoblotting. IB, immunoblot, IB*, immunoblot after stripping. To control for protein loading and transfer, blots were stripped and reprobed for β-tubulin. (**B**) The pCREB signals in each blot described in (**A**) were quantified by densitometry and normalized to β-tubulin signal in the same lane in the same blot. (**C**) HMVEC-Ls cultured to confluence in assay chambers were treated for 0.5 h with medium, FSK, or IBMX. These same chambers were then inserted into wells of 24-well plates containing either medium or IL-8 (10 ng/mL), after which calcein-AM-labeled PMNs were added to the upper compartment of each chamber. After 2 h, the contents of each lower compartment were fluorometrically assayed. Each vertical bar represents mean (+/- SEM) TEM of PMNs (%). The n for each group is indicated in each bar. * indicates significantly increased compared to the simultaneous medium controls at p < 0.05. ** indicates significantly decreased compared to IL-8 alone at p < 0.05. Click here for file
