# Supplementary material for: Bacillus anthracis-derived edema toxin (ET) counter-regulates movement of neutrophils and macromolecules through the endothelial paracellular pathway
Source: BMC Microbiol. 2012 Jan 9;12:2. doi: 10.1186/1471-2180-12-2 (PMC3277462; doi:10.1186/1471-2180-12-2)
Supplement: Additional file 1 — Figure S1. FSK and IBMX do not reproduce the ET effect on IL-8-driven TEM of PMNs at 0.5 h. (A) HMVEC-Ls were treated for 0.5 h with FSK (10 μM), IBMX (1 mM), or medium alone, and lysed. The lysates were processed for pCREB immunoblotting. IB, immunoblot, IB*, immunoblot after stripping. To control for protein loading and transfer, blots were stripped and reprobed for β-tubulin. (B) The pCREB signals in each blot described in (A) were quantified by densitometry and normalized to β-tubulin signal in the same lane in the same blot. (C) HMVEC-Ls cultured to confluence in assay chambers were treated for 0.5 h with medium, FSK, or IBMX. These same chambers were then inserted into wells of 24-well plates containing either medium or IL-8 (10 ng/mL), after which calcein-AM-labeled PMNs were added to the upper compartment of each chamber. After 2 h, the contents of each lower compartment were fluorometrically assayed. Each vertical bar represents mean (+/- SEM) TEM of PMNs (%). The n for each group is indicated in each bar. * indicates significantly increased compared to the simultaneous medium controls at p < 0.05. ** indicates significantly decreased compared to IL-8 alone at p < 0.05. [file 1471-2180-12-2-S1.PPT]

## Slide 1
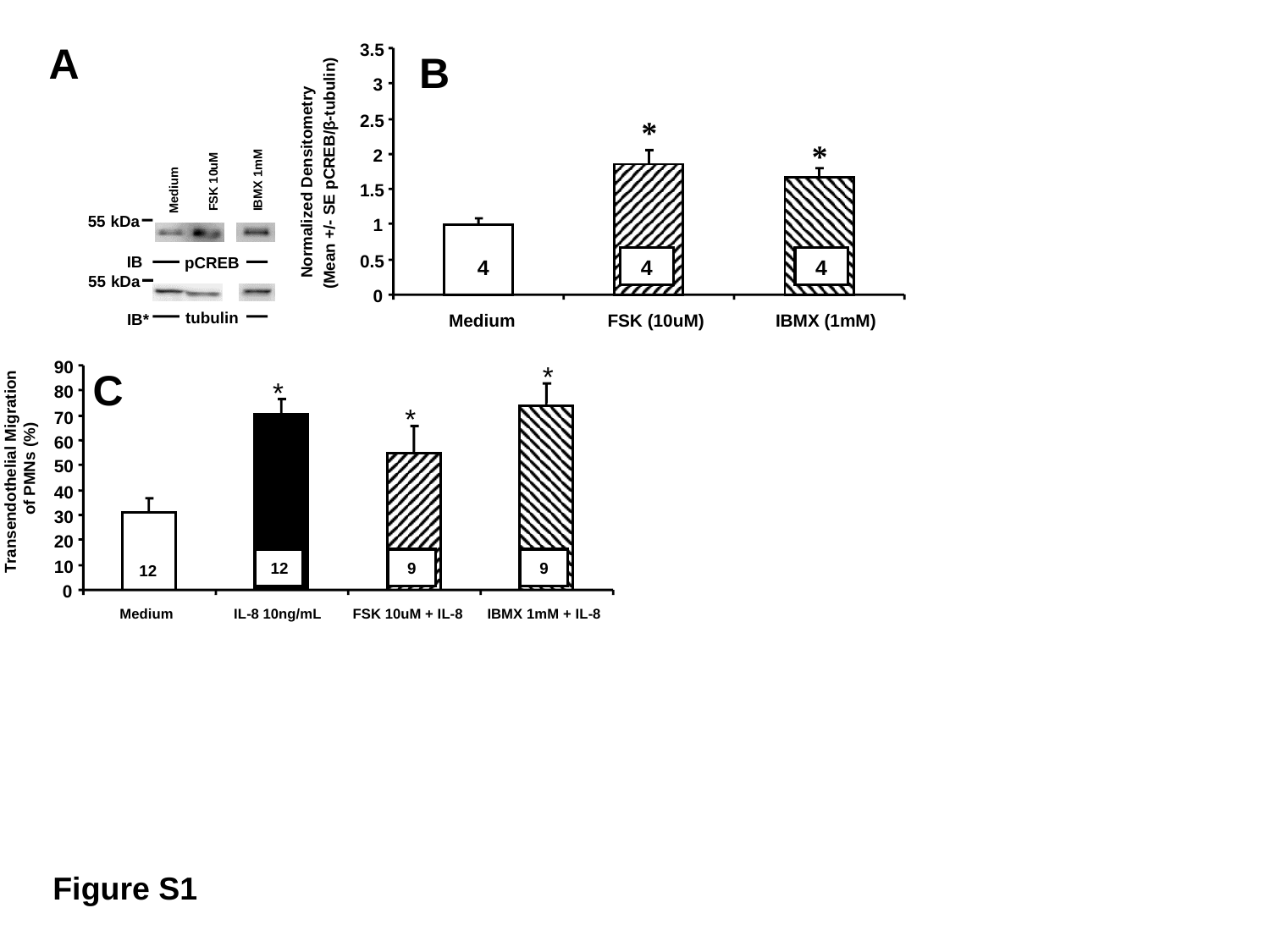

3.5
3
2.5
2
1.5
1
0.5
0
Medium
FSK (10uM)
IBMX (1mM)
)
tubulin
-
*
β
/
*
pCREB
Normalized Densitometry
SE
-
(Mean +/
4
4
4
B
A
IBMX 1mM
FSK 10uM
Medium
55
kDa
IB
pCREB
55
kDa
tubulin
IB*
90
*
C
*
80
Migration
*
70
(%)
60
50
PMNs
40
of
Transendothelial
30
20
10
12
9
9
12
0
Medium
IL
-
8 10ng/mL
FSK 10uM + IL
-
8
IBMX 1mM + IL
-
8
Figure S1
